# NOS3 27-bp and IL4 70-bp VNTR Polymorphisms Do Not Contribute to the Risk of Sickle Cell Crisis

**DOI:** 10.4274/tjh.2016.0166

**Published:** 2016-12-01

**Authors:** Henu Verma, Hrishikesh Mishra, P. K. Khodiar, P. K. Patra, L. V. K. S. Bhaskar

**Affiliations:** 1 Sickle Cell Institute Chhattisgarh, Division of Research, Raipur, India; 2 Pt. JNM Medical College, Department of Biochemistry, Raipur, India

**Keywords:** Sickle cell disease, Crisis, NOS3, IL4

## To the Editor,

A great deal of data support the direct involvement of the vascular endothelium, complex cellular interactions, and global inflammation-mediated cell activation in triggering vaso-occlusive crisis (VOC) in sickle cell disease (SCD) [1]. In the transgenic mice model for SCD, it has been shown that nitric oxide (NO) protects the mice from VOC [2]. Elevated plasma levels of certain proinflammatory cytokines support a role for cytokine-driven inflammation in SCD. The aim of the present study was to evaluate the role of the NOS3 27-bp variable number tandem repeat (VNTR) and IL4 intron-3 VNTR functional polymorphisms in the development of crisis in Indian SCD patients. The study protocol was approved by the Institutional Ethics Committee of the Sickle Cell Institute Chhattisgarh, Raipur, India. Written informed consent was obtained from the study participants. A total of 256 individuals with SCD (55.5% men) were divided into two groups based on the history of VOC. The patients hospitalized with recurrent VOC were considered as the frequent crisis (FC) group (n=140; 54.7%) and patients who had not experienced any VOC during the past 1 year were considered as the infrequent crisis (IFC) group (n=116; 45.3%). Genotyping of the NOS3 27-bp VNTR [3] and IL4 intron-3 VNTR [4] functional polymorphisms was performed and results were compared between the FC and IFC groups.

The genotype frequencies were in agreement with Hardy-Weinberg equilibrium for both the NOS3 27-bp (p=0.063) and the IL4 70-bp (p=0.614) VNTR. The genotype frequencies were not significantly different between the FC and IFC groups (Table 1). Similarly, the risk of frequent crisis was not found to be different between male and female SCD patients or between SCD patients with different HbF levels or different age groups (Table 1). Several lines of evidence suggest that there is vascular dysfunction and impaired NO bioactivity in SCD. Although no significant differences were observed in plasma NO metabolites between controls and SCD patients in the steady state, a significant reduction was noticed during VOC or acute chest syndrome [5]. Analysis of three NOS3 gene polymorphisms did not reveal a significant association with severe clinical manifestations in Brazilian SCD patients [6]. In contrast to this, in another study a significant association of NOS3 variants with VOC in SCD patients was reported [7]. However, our results indicate that the NOS3 27-bp VNTR polymorphism is not associated with the risk of frequent crises. Although the role of IL4 in SCD is controversial, increased serum IL4 levels were found in steady-state SCD patients compared to normal healthy controls [8]. Remarkably elevated levels of IL4 were noted in a VOC group compared to steady-state SCD patients and healthy controls [9]. IL4 levels correlated well with SCD status in Jamaicans, while exhibiting an ethnic difference between British and Jamaican children [10]. So far there are no published studies concerning IL4 SNPs and SCD or its complications. As these results conflict with the biological plausibility that NO and interleukin levels modulate SCD, they deserve careful interpretation and further exploration.

## Ethics

Ethics Committee Approval: The study protocol was approved by the Institutional Ethics Committee of the Sickle Cell Institute Chhattisgarh, Raipur, India, Informed Consent: Written informed consent was obtained from the study participants.

## Figures and Tables

**Table 1:**
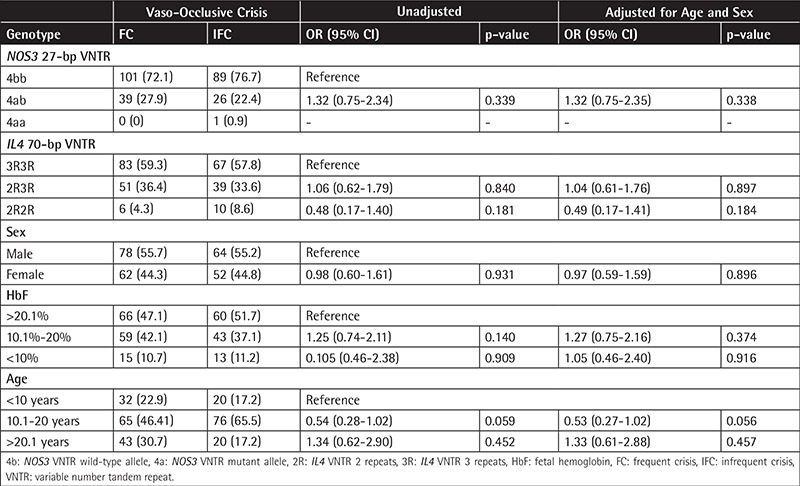
Association between NOS3 27-bp and IL4 70-bp VNTR polymorphisms and development of vaso-occlusive crisis in sickle cell disease.
